# The significance of finerenone as a novel therapeutic option in diabetic kidney disease: a scoping review with emphasis on cardiorenal outcomes of the finerenone phase 3 trials

**DOI:** 10.3389/fmed.2024.1384454

**Published:** 2024-06-14

**Authors:** Mustafa Arici, Bulent Altun, Mustafa Araz, Aysegul Atmaca, Tevfik Demir, Tevfik Ecder, Galip Guz, Dilek Gogas Yavuz, Alaattin Yildiz, Temel Yilmaz

**Affiliations:** ^1^Department of Nephrology, Hacettepe University Faculty of Medicine, Ankara, Türkiye; ^2^Department of Endocrinology and Metabolic Diseases, Gaziantep University Faculty of Medicine, Gaziantep, Türkiye; ^3^Department of Endocrinology and Metabolic Diseases, Ondokuz Mayis University Faculty of Medicine, Samsun, Türkiye; ^4^Department of Endocrinology and Metabolic Diseases, Dokuz Eylul University Faculty of Medicine, Izmir, Türkiye; ^5^Department of Nephrology, Istinye University Faculty of Medicine, Istanbul, Türkiye; ^6^Department of Nephrology, Gazi University Faculty of Medicine, Ankara, Türkiye; ^7^Section of Endocrinology and Metabolism, Marmara University School of Medicine, Istanbul, Türkiye; ^8^Department of Nephrology, Istanbul University Istanbul Faculty of Medicine, Istanbul, Türkiye; ^9^Clinics of Endocrinology and Metabolic Diseases, Florence Nightingale Hospital, Istanbul, Türkiye

**Keywords:** diabetic kidney disease, type 2 diabetes, cardiorenal risk, MR antagonists, MR overactivation, finerenone, efficacy, safety

## Abstract

This scoping review prepared by endocrinology and nephrology experts aimed to address the significance of finerenone, as a novel therapeutic option, in diabetic kidney disease (DKD), based on the biological prospect of cardiorenal benefit due to non-steroidal mineralocorticoid receptor antagonist (MRA) properties, and the recent evidence from the finerenone phase 3 program clinical trials. The importance of finerenone in slowing DKD progression was critically reviewed in relation to the role of MR overactivation in the pathogenesis of cardiorenal disease and unmet needs in the current practice patterns. The efficacy and safety outcomes of finerenone phase III study program including FIDELIO-DKD, FIGARO-DKD and FIDELITY were presented. Specifically, perspectives on inclusion of patients with preserved estimated glomerular filtration rate (eGFR) or high albuminuria, concomitant use of sodium-glucose co-transporter-2 inhibitor (SGLT2i) or glucagon-like peptide 1 receptor agonist (GLP-1 RA), baseline glycated hemoglobin (HbA1c) level and insulin treatment, clinically meaningful heart failure outcomes and treatment-induced hyperkalemia were addressed. Finerenone has emerged as a new therapeutic agent that slows DKD progression, reduces albuminuria and risk of cardiovascular complications, regardless of the baseline HbA1c levels and concomitant treatments (SGLT2i, GLP-1 RA, or insulin) and with a favorable benefit-risk profile. The evolving data on the benefit of SGLT2is and non-steroidal MRAs in slowing or reducing cardiorenal risk seem to provide the opportunity to use these pillars of therapy in the management of DKD, after a long-period of treatment scarcity in this field. Along with recognition of the albuminuria as a powerful marker to detect those patients at high risk of cardiorenal disease, these important developments would likely to impact standard-of-care options in the setting of DKD.

## 1 Introduction

Patients with diabetes are at high risk for chronic kidney disease (CKD) and the progression of CKD to end-stage kidney disease (ESKD), while the progression of both diabetes and CKD are also strongly associated with increased risk of developing cardiovascular (CV) events (1–4).

With the increase in the prevalence of type 2 diabetes (T2D) over recent decades and the limited treatment options in terms of slowing the CKD progression, CKD arising from diabetes, also known as diabetic kidney disease (DKD), has become a major contributor to the risk of cardiorenal disease progression (5–9).

The recent advances in the control of hypertension and hyperglycemia, with the use of renin-angiotensin-aldosterone system (RAAS) inhibitors such as angiotensin-converting enzyme inhibitors (ACEis) and angiotensin receptor blockers (ARBs) and, more recently, the introduction of sodium-glucose co-transporter-2 inhibitors (SGLT2is) and glucagon-like peptide 1 receptor agonists (GLP-1 RAs) has increased the chance of slowing the progression of DKD with additional CV benefits (6, 7, 10, 11). However, the continued high residual risk of progression to ESKD and CV-related morbidity and mortality in patients with DKD has motivated the further search for novel therapeutic options (6, 9, 10, 12).

In relation to improved understanding of DKD pathophysiology, and growing evidence implicating aldosterone in the pathophysiology of cardiorenal disease, the pathophysiological overactivation of the mineralocorticoid receptor (MR) has become increasingly recognized as a key driver in the progression of CKD and related morbidity (6–8, 13). Therefore, blockade of the MR has emerged as a therapeutic approach to slow the progression of CKD via anti-proteinuric, anti-inflammatory and anti-fibrotic effects (12–15).

Finerenone, a novel nonsteroidal selective MR antagonist (MRA) with a high MR affinity and a unique binding mode, was demonstrated to reduce cardiorenal injury via anti-inflammatory and anti-fibrotic mechanisms in animal models, and to significantly reduce albuminuria with a favorable safety profile comparable to placebo and less hyperkalemia than spironolactone in Phase II trials (6, 13, 16–19). Recently, data from “finerenone phase 3-program” in DKD patients on optimized RAS blockade revealed that finerenone improved the risk of CKD progression and CV events, and ameliorated albuminuria with minimal effects on parameters such as blood pressure and glycaemia (20–22).

Accordingly, representing a new frontier in RAAS inhibition with proven kidney and CV benefit in the treatment of DKD, finerenone is currently indicated to reduce the risk of kidney function decline, kidney failure, non-fatal heart attacks, CV death and hospitalization for heart failure (HHF) in patients with T2D, and is the only MRA available for this indication (8, 9, 12, 23–25).

This scoping review prepared by endocrinology and nephrology experts aimed to address the significance of finerenone among other therapeutic options to slow kidney disease progression and CV morbidity and mortality in patients with DKD, based on biological plausibility of the cardiorenal benefits provided via a non-steroidal MRA and the recent evidence from the finerenone phase 3 program clinical trials.

The main topics addressed in this paper are (a) the importance of early diagnosis and progression the risk of cardiorenal morbidity and mortality and MR overactivation in DKD, (b) unmet needs to slow the progression of cardiorenal disease in DKD (residual cardiorenal risk, incomplete RAS blockade), (c) finerenone as a novel nonsteroidal MRA (mechanism of action, inhibition of MR overactivation), (d) finerenone phase III study program (FIDELIO-DKD, FIGARO-DKD and FIDELITY efficacy and safety outcomes), (e) perspectives on finerenone phase 3 program outcomes (inclusion of patients with preserved estimated glomerular filtration rate [eGFR] or high albuminuria population, concomitant use of GLP-1 RA or SGLT2i, baseline glycated hemoglobin [HbA1c] level and insulin treatment, clinically meaningful HF outcomes), and (f) treatment-induced hyperkalemia (advantages of finerenone therapy, short-term changes in serum potassium ([K+]) and eGFR and the serum [K+] monitoring).

## 2 Diabetic kidney disease

### 2.1 Early diagnosis and progression of DKD: screening for albuminuria and eGFR

DKD is defined as structural or functional abnormalities of kidney that exist for >3 months, accompanied by eGFR of <60 mL/min/1.73 m^2^ or persistent albuminuria, in the setting of no signs or symptoms related to other primary causes of kidney damage (4, 24, 26). Although microalbuminuria (35%) or macroalbuminuria (26%) exists in the majority of T2D patients with impaired renal function, DKD may also be accompanied by normoalbuminuria (39% in total or 23% after accounting for the use of RAS inhibitors) (27).

The risks of CV events and new-onset HF increase with the severity and stage of CKD as the urinary albumin-to-creatinine ratio (UACR) exceeds 10 mg/g and the eGFR decreases below 75 mL/min/1.73 m^2^ (28–30). Hence, the timely recognition of DKD is critical to introduce measures to slow disease progression and the related CV burden (4, 8, 21, 28).

Albuminuria is considered a surrogate end point for kidney disease progression and a significant benefit in clinical outcome is predicted by a 21% to 30% reduction in UACR in patients with moderately or severely increased albuminuria (31, 32). The degree of albuminuria is associated with increased risk of CVD, CKD progression, and mortality at any GFR level (2).

**FIGURE 1:**
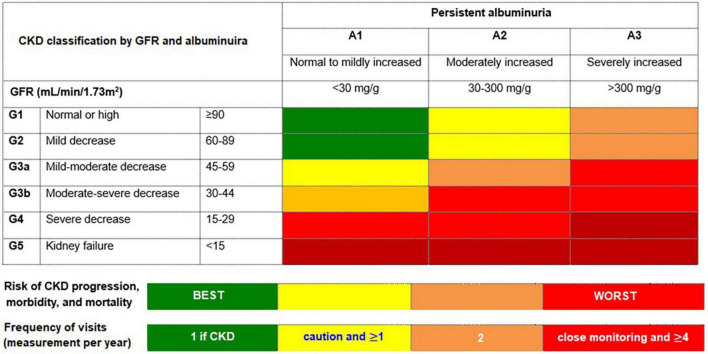
The risk of chronic kidney disease (CKD) progression according to glomerular filtration rate (GFR) and albuminuria. The GFR and albuminuria grid depicts the risk of progression, morbidity, and mortality by color, from best to worst (green, yellow, orange, red, dark red). Adapted from Kidney Disease: Improving Global Outcomes CKD Work Group (34).

Accordingly, regular assessment of both albuminuria and eGFR to identify, stage, and monitor the progression of DKD is recommended in the current screening guidelines due to their independent and synergistic association with mortality and progression to ESKD (24, 25, 33–35). Screening for albuminuria is efficiently performed by assessing UACR in a random spot urine collection (24, 25, 33–35). The Kidney Disease: Improving Global Outcomes (KDIGO) working group recommends using a more comprehensive CKD classification system that incorporates UACR (<30 mg/g [A1: normal to mildly decreased], 30–300 mg/g [A2: moderately increased], and > 300 mg/g [A3: severely increased]) at all stages of eGFR eGFR (≥ 90 [Stage 1: normal or high], 60–89 [Stage 2: mildly decreased], 45–59 [Stage 3a: mildly moderately decreased], 30–44 [Stage 3b: moderately severely decreased], 15–29 [Stage 4: severely decreased] and < 15 [Stage 5: kidney failure]) in the risk assessment (34) (Figure 1). The recommended frequency of monitoring in patients with CKD comprises 3–4 times a year for patients with UACR > 300 mg/g and eGFR < 60 mL/min/1.73 m^2^, and 2–3 times a year for patients with UACR 30–300 mg/g and eGFR 15–59 mL/min per 1.73 m^2^ and once a year in CKD patients with normoalbuminuria (< 30 mg/g) and stable disease (eGFR ≥ 60 mL/min/1.73 m^2^) (34) (Figure 1).

Due to potential risk of a high biological variability (> 20%) between measurements in urinary albumin excretion, a high or very high albuminuria is considered in case of abnormal results identified in the two of three specimens of UACR collected within a 3–6-month period (24, 25). In addition, the increased albuminuria may also accompany infection (urinary tract or systemic), hematuria or exercise (24, 25). In contrast, sudden onset or rapidly increasing albuminuria or nephrotic syndrome and rapidly decreasing eGFR suggests alternative or additional causes of kidney disease (24, 25).

Nonetheless, given that the pathologic changes related to DKD may also be present prior to development of albuminuria or low eGFR, prevention of these microvascular complications should be a management goal as early as the time of diagnosis of diabetes (25).

### 2.2 The increased risk of cardiorenal morbidity and mortality in DKD

DKD, a frequent complication that develops in up to 40% of patients with T2D, is the leading cause of ESKD and an independent risk factor of CV disease (CVD) (2–4, 36, 37). Accelerated progression to ESKD is thought to occur more frequently in patients with underlying diabetes than in patients with CKD, which is due to other etiologies (38). CKD itself also exacerbates the CV risk (28, 37), while T2D patients with CKD are at higher risk for CV morbidity than for the progression to ESKD and three times more likely to die from a CV cause than those without CKD (3, 8, 39–42).

### 2.3 The role of MR overactivation in the pathogenesis of cardiorenal disease

As a member of a superfamily of nuclear hormone receptors, MR is predominantly expressed in the heart, kidneys, vasculature, brain, gut and myeloid cells (43). The role of MR gene expression in controlling the fluid, electrolyte and hemodynamic homeostasis is better recognized than the role of MR overactivation in stimulating inflammation and fibrosis and the progression to end-organ damage in cardiorenal disease (6, 13, 16, 41).

Notably, growing evidence supports a pathophysiological role for MR overactivation, as driven by metabolic, hemodynamic or inflammatory and fibrotic factors, in occurrence of progressive kidney and CV dysfunction during DKD (13, 14, 18, 42). In patients with CKD and diabetic nephropathy, MRAs, when added to the standard treatment (ACE inhibitor/ARB therapy), was reported to yield reduction in albuminuria (23% to 61%), regardless of the blood pressure changes (44, 45). Hence, MR blockade via MRAs has become a promising pharmacological target for preserving organ function particularly in patients with DKD, which is of critical importance in terms of slowing the progression of CKD and reducing CV morbidity and mortality (6, 7, 13–15, 41, 43, 46). Given the higher effectiveness of interventions in early CKD in delaying kidney disease progression, amelioration of inflammation and fibrosis at the earliest possible stage is considered the most effective strategy (6, 34, 47).

## 3 Unmet needs to slow the progression of cardiorenal disease in DKD

### 3.1 The residual cardiorenal risk despite the latest glucose-lowering therapies

Slowing the progression of DKD is critical for reducing risk of cardiorenal morbidity and mortality (25). High levels and variability of blood glucose and blood pressure and the albuminuria are important risk factors for DKD (25, 48). The mainstay therapeutic approaches in slowing the progression of CKD in T2D involve the control of hyperglycemia and the use of RAS blockers such as ACEi or ARB for albuminuria with or without hypertension (6, 11, 26, 34, 48, 49).

Intensive glucose control (HbA1c levels < 7%) was associated with reduced risk of incident albuminuria and DKD onset in several clinical trials. In contrast, its efficacy in slowing the progression of DKD has not been shown, and no solid evidence exists on the link between glycemic control and disease outcomes, particularly in the case of moderate to severe DKD (25, 50–54).

More recently, the use of an SGLT2i, one of the latest glucose-lowering therapies with benefits beyond blood glucose control, in addition to ACEi or ARB has become a guideline-recommended strategy for the reduction of cardiorenal risk in T2D patients with albuminuria > 30 mg/g and eGFR ≥ 20 mL/min/1.73 m^2^ (11, 25, 26, 55). However, despite the use of RAS inhibitors (ACEi or ARB) plus concomitant SGLT2i, CREDENCE and DAPA-CKD trials showed that CKD progression or kidney failure still occurred in approximately 10% of patients (56, 57), indicating that patients with CKD and T2D remain to be at considerable risk of CKD progression and CV events (4, 6, 10, 36).

Although, GLP-1 RAs are also recommended in clinical guidelines as another new glucose-lowering therapy with additional beneficial effects on CV outcomes, particularly in patients with DKD patients and eGFR ≥ 15 mL/min/1.73 m^2^ and to reduce risks of atherosclerotic CVD (ASCVD), macroalbuminuria, and eGFR decline; their kidney protection capacity is yet to be defined (25, 58).

### 3.2 Inability to offer complete blockade of RAAS

While the RAS inhibitors are the basis of therapy in patients with DKD and the ACEi or ARB have decreased proteinuria, progression of CKD and mortality, there remains a significant residual risk for these events (13, 15, 16, 34, 59). In order to overcome this risk, prior trials of dual RAS blockade have unfortunately failed to show CV or kidney protection in patients with DKD (60–63). These findings suggest that how the RAAS is blocked is important in achieving effective and safe cardiorenal protection (22).

The available steroidal MRAs (spironolactone and eplerenone) are used for hypertension and HF treatment and are known to reduce proteinuria added to ACEi or ARBs, but not indicated in patients with reduced renal function or DKD due to concerns of hyperkalemia (6, 41, 59). Although guidelines recommend spironolactone as optimal fourth-line therapy of resistant hypertension with the strongest endorsement (class IA) for the treatment of HF with reduced ejection fraction (HFrEF), this indication is restricted only to patients with eGFR > 45 mL/min/1.73 m^2^ and serum [K+] ≤ 4.5 mEq/L (41, 64, 65). Hence, the risk of hyperkalemia considerably limits the widespread use of these lifesaving steroidal MRAs in clinical practice, not only in patients with impaired kidney function but also in cases where MRAs are not contraindicated (41, 66). In this regard, whether the steroidal MRAs are also effective in slowing the progression of kidney injury in patients with DKD remains uncertain with a lack of clinical trial evidence in this high-risk population (6, 41, 67, 68).

Overall, the inability to offer a complete blockade of RAS affects clinical outcomes, increasing the long-term risk for adverse cardiorenal events and mortality (42, 67). This necessitates novel treatments for DKD that would improve the pathways of inflammation, fibrosis and oxidative stress to slow the progression of DKD and to reduce residual cardiorenal risk (7). In this regard, several novel non-steroidal MRAs with higher potency and selectivity and a more favorable side-effect profile (i.e., esaxerenone, apararenone and finerenone) have been developed over the last decade, allowing the testing of MRA safety and efficacy in large populations of patients (6–8, 10, 12, 13, 41, 59). Hence, in addition to the RAS blockers and SGLT2is, a novel class of agents called non-steroidal MRAs are now available as potent, selective and cardioprotective MRAs with a favorable safety profile (8, 12, 15, 69).

## 4 Finerenone: a novel nonsteroidal MRA

Finerenone is a third-generation, nonsteroidal MRA with a high MR affinity and a unique binding mode enabling its potency, selectivity, and nuclear cofactor recruitment (13). The association of finerenone with reduction in the risk of substantial GFR decline, kidney failure, HF and ASCVD events, and related mortality has been demonstrated in a broad T2D population with a varied renal dysfunction ranging from microalbuminuria to advanced CKD (20–22). Accordingly, finerenone is a novel selective nonsteroidal MRA recommended for kidney and heart protection in T2D patients with an eGFR ≥ 25mL/min/1.73 m^2^, normal serum [K+], and albuminuria (UACR ≥ 30 mg/g) on a maximum tolerated dose of a RAS inhibitor (24, 25).

### 4.1 Mechanism of action

While the steroidal MRAs (spironolactone and eplerenone) show a partial agonistic effect on cofactor recruitment, finerenone acts as a bulky-passive MR antagonist and impairs MR signaling at various levels via blocking MR-mediated sodium reabsorption and MR overactivation (13, 41, 70–72).

**FIGURE 2:**
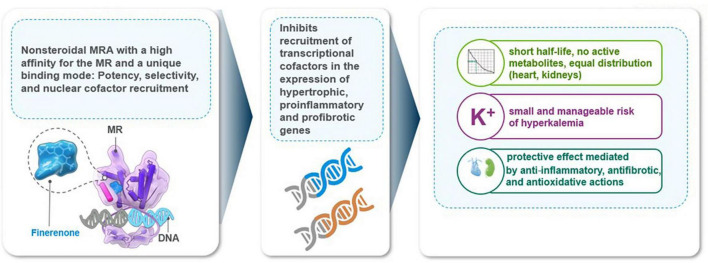
Finerenone mechanism of action.

Finerenone has important advantages such as greater MR selectivity (vs. spironolactone) and higher receptor binding affinity (vs. eplerenone) and is at least equally potent compared with spironolactone (16, 69, 71). The unique MR binding enables the inhibitory action of finerenone on expression of hypertrophic, proinflammatory and profibrotic genes, regardless of the presence or absence of aldosterone (17, 41, 43, 70, 72, 73). While steroid MRAs exhibit more significant accumulation in the kidneys than in the heart, finerenone shows the equal distribution in the heart and kidney, and the clearance of finerenone is mainly mediated through non-renal routes of elimination and without biologically active metabolites (7, 13, 41) (Figure 2).

The distinct mode of MR antagonism accompanied with transcriptional cofactor recruitment, a short plasma half-life with no active metabolites, and the equal distribution to heart and kidneys are considered to enable finerenone to have minimal effects on serum [K+] (13, 17, 69–73) (Figure 2).

The preclinical evidence indicates that finerenone is effectively slows end-organ damage in cardiorenal disease and blocking complementary pathogenic mechanisms with its anti-inflammatory, antifibrotic and antioxidative stress properties (7, 8, 17, 69–73). Hence, unlike RAS inhibitors and the SGLT2is, the reno-protective effect of finerenone is suggested to be mediated by its anti-inflammatory, antifibrotic and antioxidative actions rather than the altered renal hemodynamics or tubuloglomerular feedback (7, 8, 14, 17, 69–73) (Figure 2).

### 4.2 Inhibition of MR overactivation by finerenone

In fact, both steroidal MRAs and novel, nonsteroidal MRAs can inhibit MR overactivation and reduce its hazardous effects by reducing proinflammatory and profibrotic gene expression (6, 13). Available steroidal MRAs (spironolactone and eplerenone) has limited use in patients with T2D and CKD due to the risk of complications such as antiandrogenic side effects and hyperkalemia (6, 8, 43). Moreover, an increase in HbA1c levels was reported in patients receiving spironolactone (74).

Data from the phase II ARTS program (ARTS, ARTS-HF and ARTS-DN) in patients with HF and DKD revealed that finerenone reduced albuminuria and N-terminal pro B-type natriuretic peptide with a lower risk of hyperkalemia when compared to steroidal MRAs (19, 43, 75, 76). This implicates that the complications restricting the use of steroidal MRAs (hyperkalemia or reductions in kidney function) may not be the limiting factors for the use of finerenone (19, 43, 75, 76). Finerenone results in MR blockade that is not inferior to spironolactone and more selective than eplerenone with more effective reduction in inflammation, fibrosis, cardiac hypertrophy and proteinuria, as reported in rodent kidney models (6, 16–18, 72).

**FIGURE 3:**
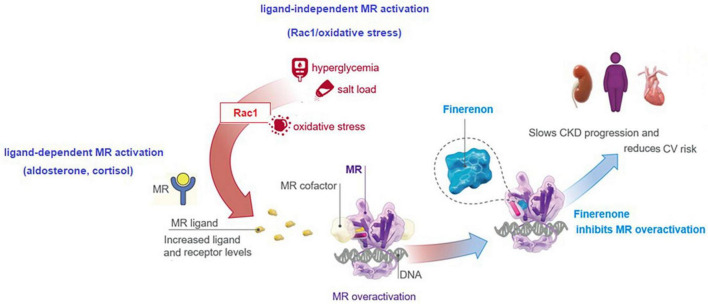
Inhibition of MR overactivation by finerenone.

Increasing evidence implicates a multidirectional interaction and upregulation between aldosterone, the MR, and Ras-related C3 botulinum toxin substrate 1 (Rac1), as driving forces in the onset of chronic interstitial nephritis and progression to fibrosis in CKD and DKD (77). Increased MR expression in the setting of DKD is mediated by the increased MR ligand (aldosterone, cortisol) and receptor levels, and the ligand-independent MR activation via a cross-talk between the Rac1/oxidative stress and MR (77, 78). By blocking MR-mediated sodium reabsorption and MR overactivation, finerenone was reported to inhibit inflammatory, fibrotic, oxidative and hypertrophic processes in preclinical models, explaining its kidney and CV benefits (8, 13, 17, 18, 72, 73). This translates to beneficial actions of finerenone in the setting of DKD in terms of tissue effects (inflammation, fibrosis, oxidative stress, hypertrophy) and the clinical effects (albuminuria, eGFR, hypertension, kidney outcomes, CV outcomes) (8, 13, 20–22) (Figure 3).

## 5 Finerenone phase III study program: FIDELIO-DKD and FIGARO-DKD trials and FIDELITY meta-analysis

### 5.1 Design and study protocols

The finerenone phase III program, the largest cardiorenal outcomes program in T2D patients with CKD, evaluated the effect of finerenone vs. placebo on top of maximum tolerated RAS inhibition, on kidney and CV outcomes in more than 13,000 patients with mild-to-severe CKD and T2D worldwide (20–22). The program comprised two randomized, double-blind, placebo-controlled phase 3 trials with complementary protocols, namely the FIDELIO-DKD (FInerenone in reducing kiDnEy faiLure and dIsease prOgression in Diabetic Kidney Disease) and FIGARO-DKD (FInerenone in reducinG cArdiovascular moRtality and mOrbidity in Diabetic Kidney Disease) trials (20, 21).

FIDELIO-DKD investigated the efficacy and safety of finerenone in delaying CKD progression in advanced CKD in approximately 5,700 patients with CKD and T2D (20). In contrast, FIGARO-DKD evaluated the efficacy and safety of finerenone in reducing CV morbidity and mortality in earlier stages of CKD in approximately 7,400 patients with CKD and T2D (21).

**FIGURE 4:**
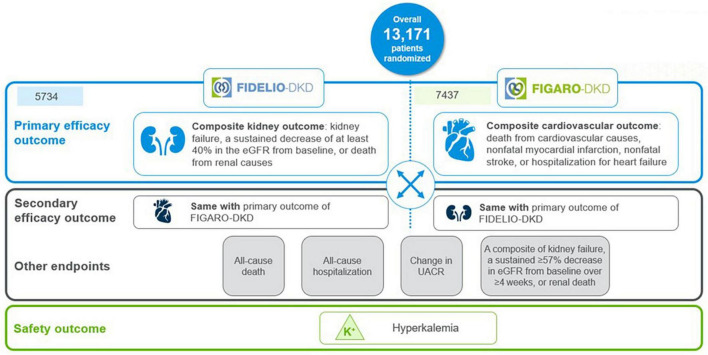
FIDELIO-DKD and FIGARO-DKD endpoints.

Kidney outcome (composite of time to first occurrence of kidney failure, a sustained decrease of at least 40% in the eGFR from baseline, or death from renal causes) was the primary endpoint in FIDELIO-DKD and the secondary composite outcome in FIGARO-DKD. The CV outcome (composite of death from CV causes, nonfatal myocardial infarction, nonfatal stroke, or HHF) was the primary endpoint in FIGARO-DKD and the secondary composite outcome in FIDELIO-DKD (6, 20, 21) (Figure 4).

FIDELITY (The FInerenone in chronic kiDney diseasE and type 2 diabetes: Combined FIDELIO-DKD and FIGARO-DKD Trial programme analYsis), was a pooled analysis of FIDELIO-DKD and FIGARO-DKD trials, aimed to provide more robust estimates of finerenone efficacy and safety across the spectrum of patients with CKD and T2D (22). Main time-to-event efficacy outcomes were a composite of CV death, non-fatal myocardial infarction, non-fatal stroke, or HHF, and a composite of kidney failure, a sustained ≥ 57% decrease in eGFR from baseline over ≥ 4 weeks, or renal death (22).

### 5.2 Efficacy outcomes

**FIGURE 5:**
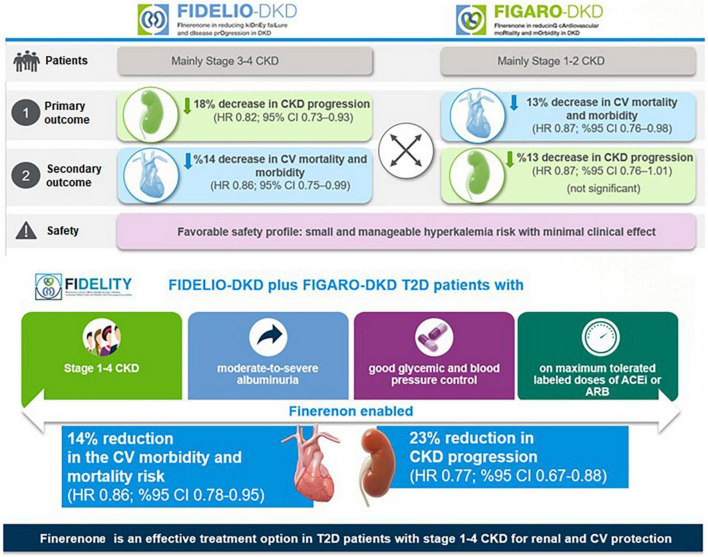
FIDELIO-DKD, FIGARO-DKD, and FIDELITY outcomes.

In the FIDELIO-DKD trial, finerenone significantly reduced the primary composite kidney outcome by 18% [Hazard ratio (HR) 0.82, 95% confidence interval CI) 0.73–0.93, *p* = 0.001] and the key secondary composite CV outcome by 14% (HR 0.86, 95% CI 0.75–0.99, *p* = 0.03) compared with placebo in patients receiving optimized RAS inhibitor therapy (20) (Figure 5).

Hence, results from FIDELIO-DKD indicated that finerenone, when combined with the optimized RAS blockade therapy, offers a new effective treatment option in DKD in terms of slowing the progression of DKD and primary and secondary prevention of CV events (20, 79).

In FIGARO-DKD trial, the primary composite CV outcome was reduced by 13% (HR 0.87, 95% CI, 0.76–0.98, *p* = 0.03), with the benefit driven primarily by a lower incidence of HHF (HR 0.71; 95% CI, 0.56–0.90), while the secondary composite kidney outcome was reduced non-significantly by 13% (HR 0.87, 95% CI, 0.76–1.01) (21) (Figure 5).

Hence, FIGARO-DKD showed the association of finerenone with a lower risk of the CV morbidity and mortality, particularly in terms of a lower incidence of HHF in patients with DKD (stage 2–4 CKD with moderately elevated albuminuria or stage 1 or 2 CKD with severely elevated albuminuria) (21). Although the kidney composite outcomes including at least 40% decrease in eGFR from baseline were similar for finerenone in both trials, the significance was not achieved in the FIGARO-DKD (21). However, in both trials, when the kidney outcome was considered as at least 57% decrease in the eGFR (a more sensitive surrogate outcome for kidney failure than a decrease of ≥ 40% in the eGFR), it was lower in the finerenone vs. the placebo group (20, 21).

The FIDELITY analysis showed robust evidence of both CV and kidney protection with finerenone vs. placebo, including a 30% risk reduction in doubling (> 57% reduction) in serum creatinine and a 20% risk reduction in ESKD, and significant 23% relative risk (RR) reduction of the composite kidney outcome (5.5% vs. 7.1%, HR 0.77; 95% CI, 0.67–0.88; *p* < 0.001). In addition, there was a 14% risk reduction in composite cardiovascular outcome (12.7% vs. 14.4%, HR 0.86, 95%CI, 0.78–0.95; *p* = 0.0018), primarily driven by a reduction in HF hospitalization, (22). The blood pressure changes in FIDELITY were modest (2.5 mmHg mean systolic blood pressure reduction) but cannot explain the CV and renal protective effects of finerenone (22) (Figure 5).

### 5.3 Safety outcomes

Finerenone, via MR antagonism, is expected to result in increased serum [K+] (13). Both FIDELIO-DKD (18.3% vs. 9.0%) and FIGARO-DKD (10.8% vs. 5.3%) revealed that hyperkalemia-related adverse events were twice as frequent with finerenone compared with placebo in (20, 21). However, total incidence of treatment-emergent adverse events was similar between the finerenone and placebo groups along with the low frequency of clinically relevant hyperkalemia-related adverse events and hyperkalemia-related permanent treatment discontinuation (20, 21).

Consistent with a higher mean eGFR of patients in the FIGARO-DKD than in the FIDELIO-DKD trial (68 vs. 44 mL/min/1.73 m^2^), the incidence of hyperkalemia with finerenone was lower (10.8% vs. 18.3%) in the FIGARO-DKD, despite the longer median follow-up (3.4 vs. 2.6 years) (20, 21). FIDELTIY revealed that finerenone and placebo arms were similar in terms of overall safety outcomes and the rates of hyperkalemia leading to permanent treatment discontinuation (1.7% vs. 0.6%, respectively) (22) (Figure 5). The discontinuation rates for finerenone and placebo were 0.9 and 0.4%, respectively, in patients with an eGFR ≥ 60. Apart from hyperkalemia, finerenone revealed no clinically significant side effects which were reported with steroidal MR agonists (i.e., gynecomastia, impotence and menstrual irregularities),(20–22).

## 6 Perspectives on finerenone phase 3 program outcomes

The current standard of care in DKD is mainly based on glycemic control and blood pressure management, with inflammation and fibrosis remain to be largely unaddressed (8). Hence, SGLT2i and GLP-1 RA are currently recommended for T2D patients with or at high risk for ASCVD, HF, and/or CKD given their proven CV and renal benefit (9, 24). Following the evidence regarding the efficacy of finerenone in reducing the occurrences of cardiorenal outcomes from trials in the finerenone phase 3 program (20, 21), finerenone as a new highly effective therapy, in addition to SGLT2i and GLP-1 RA, has also become involved in the recent clinical guidelines which update their recommendations to include the finerenone for patients with T2D and CKD treated with maximum tolerated doses of ACEi or ARBs to improve CV outcomes and reduce the risk of CKD progression (24, 25).

### 6.1 Inclusion of patients with preserved eGFR or high albuminuria

**FIGURE 6:**
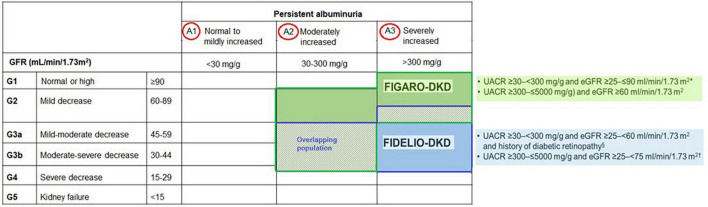
Finerenone population by GFR and albuminuria categories.

Finerenone program included previously understudied patient groups, such as those with high albuminuria (UACR 30–300 mg/g) or very high albuminuria (UACR > 300 mg/g) and eGFR > 60 mL/min/1.73 m^2^ (6). In total, 39.9% and 31.2% of patients included in both trials corresponded to patients with preserved eGFR and high albuminuria, respectively, who are often excluded from DKD trials, while moderate, high and very high KDIGO risk scores (a combination of eGFR and UACR categories) were noted in 10, 41.1, and 48.3% of patients, respectively (6) (Figure 6).

Given that albuminuria, even within the normal upper range, is an independent risk marker for CV and all-cause mortality, this high-risk CV population provides further insight into the potential benefit of finerenone for reducing CV outcomes in patients with earlier stages of CKD (6, 28).

The FIDELITY data revealed a 30% reduction in the risk of a sustained ≥ 57% decrease in eGFR over optimized ACEi or ARB therapy, as well as a 20% relative risk reduction in ESKD with finerenone vs. placebo (22). This seems to be of critical importance regarding the potential reduction in the requirement for dialysis, while the reduction in the risk of clinically meaningful CV and kidney outcomes across the spectrum of CKD emphasize the potential of early treatment onset prior to progression of CKD in achieving improved outcomes in this patient population (22).

### 6.2 Concomitant use of GLP-1 RA or SGLT2i

• In FIDELIO-DKD and FIGARO-DKD trials, ∼7% of patients were on SGLT2is and GLP-1 RA therapy at baseline, while the CV and kidney benefits of finerenone were found to be similar in patients with vs. without concomitant SGLT2i or GLP-1 RA therapy (6, 22).
• In a subgroup analysis of FIDELIO-DKD, regarding the effect of GLP-1 RA use (6.9% of the total FIDELIO-DKD population) on the treatment effect of finerenone (80), the addition of finerenone to a GLP-1 RA was associated with further reduction in albuminuria, while reduced kidney disease progression and CV events were noted with finerenone vs. placebo, regardless of GLP-1 RA treatment. Concomitant GLP-1 RA treatment had no effect on clinical benefits and safety profile of finerenone (80), emphasizing that finerenone has a kidney protective effect in patients with ongoing GLP-1 RA, a treatment that reduces UACR (11, 80, 81).

In a subgroup analysis of FIDELIO-DKD, regarding the effect of SGLT2i use (4.6% of the total FIDELIO-DKD population) on the treatment effect of finerenone (82), finerenone improved UACR reduction by 25% in patients receiving concomitant SGLT2i, indicating the efficacy of finerenone in patients with ongoing SGLT2i, a drug known to reduce UACR (82). The benefits of finerenone on kidney and CV outcomes were considered to be irrespective of the use of SGLT2i, while fewer hyperkalemia events were observed in finerenone-treated patients with vs. without concomitant SGLT2i (8.1% with vs. 18.7% without) (82). The therapies that enable further lowering of UACR when used as add on to SGLT2is are considered likely to provide additional kidney and CV benefits beyond the use of SGLT2i alone (31, 82). In this regard, given its association with lower rate of hyperkalemia-related AEs and reduction in UACR, add-on SGLT2i and/or finerenone seems to represent a valuable cardiorenal protective therapy in DKD patients (8, 21, 22, 55, 82).

Nonetheless, the potential additive effect of the combination of finerenone on top of SGLT2i or GLP-1 RA, possibly due to their distinct mechanisms of action, should be further investigated for the efficacy and safety of using these agents in combination in patients with DKD (21, 22). There are ongoing RCTs such as the CONFIDENCE (COmbinatioN effect of FInerenone anD EmpaglifloziN in participants with CKD and type 2 diabetes using a UACR Endpoint) study investigating whether dual finerenone and the SGLT2i empagliflozin therapy is superior to either drug alone in reducing UACR, and thus may contribute to slowing disease progression along with long-term benefits (83).

Notably, in a network meta-analysis of 18 RCTs involving 51,496 patients, the relative efficacy of three drugs (finerenone, SGLT2i, and GLP-1 RA) on CV and renal outcomes in patients with DKD was evaluated (9). Both finerenone and SGLT2i reduced the risk of major adverse CV events (MACE), renal outcome and HHF, while SGLT2i also significantly reduced risks of all-cause death and CV death and GLP-1 RA revealed only a lower risk of MACE (9). In addition, SGLT2i was associated with a stronger effect on the renal outcome and HHF in comparison to both finerenone (RR 1.29, 95% CI, 1.13–1.47 and RR 1.31, 95% CI, 1.07–1.61, respectively) and GLP-1 RA (RR 1.36, 95% CI, 1.16–1.59 and RR 1.49, 95% CI, 1.18–1.89, respectively) (9). The authors concluded that finerenone has the advantage of reducing MACE risk just as well as SGLT2i. at the same time, SGLT2i outperforms finerenone in terms of reducing the risk of renal outcome and HHF, possibly related to the unique potency of SGLT2i in reducing blood glucose, losing weight, controlling blood pressure besides reducing oxidative stress, improving renal ultrafiltration and hypoxia and reducing uric acid (9, 84).

### 6.3 Baseline HbA1c level and insulin treatment

In a subgroup analysis of FIDELIO-DKD population assessing the efficacy of finerenone with respect to baseline HbA1c levels (< 7.5% in 49.3% of patients) or insulin use (64.1% of the total population), finerenone was found to reduce the risk of the kidney composite outcome and CV composite outcome incidence, independent of baseline HbA1c level and insulin use (54). Hence, in contrast to other approved therapies aiming to reduce cardiorenal risk, finerenone is considered to delay CKD progression and reduce CV events in patients with DKD, irrespective of baseline HbA1c level and insulin use, and without affecting HbA1c levels (54).

### 6.4 Clinically meaningful HF outcomes

In FIGARO-DKD trial, despite the exclusion of patients with HFrEF, HHF was a key driver of the primary outcome, which is the first indication that a nonsteroidal MRA may provide benefit in a population with DKD and without HFrEF, and thus in patients at risk of HF or early-stage HF (21). The findings also emphasize the likelihood of finerenone to represent an advance in the prevention and management of HF and reduce health care burdens, since DKD patients with new-onset or preexisting HF are at very high risk for hospitalization and mortality (21, 85).

In the prespecified analyses of the FIGARO-DKD trial regarding the effects of finerenone on the incidence of new-onset HF and the benefit of finerenone by baseline history of HF (7.8% of total FIGARO-DKD population) (86), finerenone reduced the new-onset HF versus placebo (HR 0.68, 95% CI, 0.50–0.93, *p* = 0.0162), and the effects of finerenone on improving HF outcomes (18% lower risk of CV death or first HHF, a 29% lower risk of first HHF and a 30% lower rate of total HHF) were not affected by a history of HF (86). These analyses indicate that finerenone reduces new-onset HF and improves other HF outcomes in patients with DKD, irrespective of a history of HF (86).

The FIGARO-DKD trial included patients at high CV risk, and less advanced kidney disease than those in FIDELIO-DKD (20, 21, 86). Notably, the magnitude of risk reduction for total HHF was smaller in FIDELIO-DKD vs. FIGARO-DKD (14 vs. 30%) (20, 21, 86). Given that in patients with DKD and HF, mortality and hospitalization rates increase with CKD severity (87), the stronger effect of finerenone on HF outcomes in the FIGARO-DKD trial may emphasize that initiating treatment at earlier stages of the disease may be more beneficial for this patient population (86). Also, FIGARO-DKD included patients with less advanced CKD than other trials in the setting of DKD and the CV benefits of finerenone therapy were consistent across categories of baseline UACR and eGFR (21). Hence, using UACR and HF screening to identify patients at risk is considered critical to the cardiorenal disease burden in this patient population (21, 22, 86). Moreover, in the subgroup of patients receiving an SGLT2i at baseline, a greater effect on the composite outcome of CV death and HHF was observed in the finerenone plus SGLT2i group vs. SGLT2i alone group, suggesting an increasing treatment benefit with a combined use of finerenone plus SGLT2i (86).

## 7 Treatment-induced hyperkalemia

### 7.1 Advantages of finerenone therapy

Previous kidney-outcome trials in DKD patients that target dual RAS blockade revealed a lack of efficacy and increased risk of adverse events (i.e., acute kidney injury [AKI], hypotension and hyperkalemia), possibly in relation to the inhibition of two proximal targets in the RAS cascade (60, 61). In the FIDELIO-DKD trial, while finerenone had a higher overall risk of hyperkalemia than placebo, hyperkalemia-based treatment discontinuation was infrequent (2.3%) and markedly lower than reported in trials of dual RAS blockade (4.8% with a direct renin inhibitor plus an ACEi or ARB and 9.2% with dual ACEi-ARB therapy), despite FIDELIO-DKD had no protocol recommendations to restrict dietary potassium or potassium supplements in contrast to studies of dual RAS blockade (20, 60, 61).

In a post hoc safety analysis of FIDELIO-DKD trial on incidences and risk factors for hyperkalemia with finerenone vs. placebo, over 2.6 years’ median follow-up, while finerenone was associated with higher rate of treatment-emergent mild hyperkalemia (21.4 vs. 9.2%, respectively) and moderate hyperkalemia (4.5 vs. 1.4%, respectively), it is considered a manageable hyperkalemia risk (88). Independent risk factors for mild hyperkalemia included a higher baseline serum potassium, lower eGFR and increased UACR, while diuretic or SGLT2i use reduced the risk (88). Accordingly, this sub-analysis emphasized a solid and robust relationship between higher UACR with increased hyperkalemia, which is a less widely recognized risk factor for hyperkalemia than the lower eGFR (< 45 mL/min/1.73 m^2^) and higher baseline [K+] (> 4.5 mmol/L) (88–91).

The FIDELIO-DKD trial revealed a more favorable tolerability profile with finerenone than previously reported with spironolactone in the AMBER (Spironolactone With Patiromer in the Treatment of Resistant Hypertension in Chronic Kidney Disease) trial, in terms of lower rates of treatment discontinuation due to hyperkalemia (2.3% with finerenone over 2.6 years, 23.0% with spironolactone and 6.8% with spironolactone plus potassium-binder patiromer over 12 weeks) (92). Hence, while the risk of hyperkalemia with finerenone is valid, this risk is considered minor and manageable, with no adverse effect of baseline serum [K+] categories on cardiorenal protection offered by finerenone (41).

In a head-to-head study with spironolactone and finerenone in patients with chronic HF and mild-to-moderate CKD, finerenone had comparable effects on efficacy markers and cardiac biomarkers of hemodynamic stress and albuminuria and was associated with a significantly less increase in serum [K+] (mean 0.04-0.30 vs. 0.45 mEq/L) and lower incidence of hyperkalemia (5.3% vs. 12.7%) (75). The phase IIb ARTS (Mineralocorticoid Receptor Antagonist Tolerability Study) trial in patients with HFrEF and mild-stage CKD revealed that finerenone (10 mg once daily) vs. spironolactone (25 to 50 mg once daily) yielded a similar reduction in N-terminal pro-B-type natriuretic peptide but a lower hyperkalemia rate (4.5% versus 11.1%, respectively) (93). The ARTS-HF trial on the comparison of finerenone vs. eplerenone in patients with worsening HFrEF and CKD and/or T2D, finerenone was associated with a less increase in serum K+ (0.119–0.202 vs. 0.262 mEq/L) and a lower rate of having a K+ increase to ≥ 5.6 mmol/L at any time during the study (3.6–3.8 vs. 4.7%) (76). In the ARTS-DN (Mineralocorticoid receptor antagonist tolerability study- diabetic nephropathy), in patients with diabetic nephropathy under RAS blockade, finerenone induced a significant dose-dependent reduction in proteinuria (50% reduction in proteinuria in 40% of patients with 20 mg/day), along with a low rate of hyperkalemia (K+ > 5.6 mEq/L) leading to treatment discontinuation (1.5% vs. 0.0% in placebo) (19).

Notably, the short half-life of finerenone (2–3 h in patients with CKD) and lack of active metabolites are important significant advantages that enable finerenone-associated hyperkalemia to be effectively managed by treatment interruption, as demonstrated in FIDELIO-DKD (13, 88, 94). In contrast, the long half-life and multiple active metabolites of spironolactone along with its kidney versus heart tissue distribution (6:1 vs. 1:1 for finerenone) indicates that spironolactone interacts with the MR in a different manner than finerenone (13, 88, 94).

### 7.2 Short-term changes in serum [K+] and eGFR after the start of treatment

The post hoc safety analysis of FIDELIO-DKD revealed that short-term increases in serum [K+] after the onset of treatment were predictive of subsequent risk of hyperkalemia, similarly in placebo and finerenone groups (88). However, for “any given increase” vs. “no change” in serum [K+] from baseline to month 4, the increased risk of hyperkalemia was smaller with finerenone than with placebo (88). This is suggested to be related to the manageable nature of the finerenone-related hyperkalemia (via treatment interruption, dose reduction, or use of diuretics or potassium binders), but higher likelihood of placebo-related hyperkalemia to be associated with conditions reducing the renal potassium secretion (i.e., AKI, tubulointerstitial inflammation, or obstruction) which are less amenable to treatment interventions (88, 89).

In addition, in both groups, the short-term decreases in eGFR after treatment onset were associated with an increased risk of hyperkalemia, whereas the magnitude of the increased risk for “any given reduction” vs. “no change” in eGFR was smaller with finerenone than placebo (88). It is explained by the hemodynamic (in contrast to tubular cause) nature of the decrease in eGFR induced by finerenone as provoked by natriuresis or modest BP reduction, which is less likely to adversely impact the ability to secrete potassium (88). Hence, temporary finerenone discontinuation and dose reduction is considered likely to restore eGFR and normalize serum [K+] (88, 92). Furthermore, since finerenone slows eGFR decline vs. placebo, this may also reduce the risk of subsequent hyperkalemia (20).

Indeed, some pharmacodynamic effects of MRAs (i.e., blood pressure control or serum [K+] changes) are considered to occur after a significant drug exposure over a long period (long half-life), while others (i.e., anti-inflammatory, antihypertrophic, and antifibrotic effects) appeared to occur after a relatively short drug exposure (short half-life), as induced by different signaling cascades (75, 88). Notably, 5 mg twice daily vs. 10 mg once daily doses of finerenone were associated with a more remarkable rise in serum [K+] but similar reductions of N-terminal pro–B-type natriuretic peptide or albuminuria, emphasizing the significance of both pharmacokinetics and physiology when considering hyperkalemia rates (75, 88).

### 7.3 Serum [K+] monitoring

**FIGURE 7:**
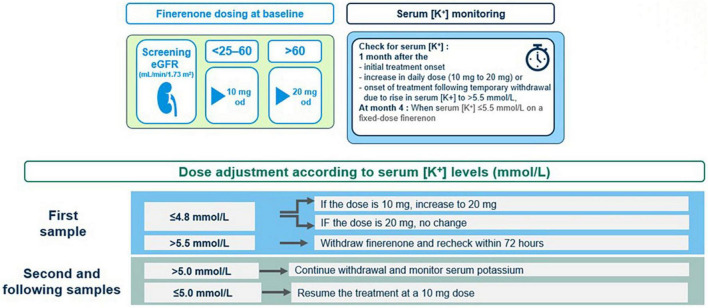
Serum [K+] monitoring and finerenone dose adjustment.

In clinical practice, physicians often reduce the dose of RAS inhibitors when serum [K+] rises above 5.0 mmol/L (95). However, in finerenone phase 3 program, RAS inhibitor dose reduction was not permitted and finerenone was continued with no dose adjustments in patients with a serum [K+] of 5.0–5.5 mmol/L. Finerenone was temporarily withheld for serum [K+] > 5.5 mmol/L, and the treatment was resumed (at the 10-mg dose) when serum [K+] has become ≤ 5.0 mmol/L (22, 88) (Figure 7).

The scheduled serum [K+] assessment was at 1 month after the onset of treatment, followed by the second assessment at month 4 and at 4-monthly intervals thereafter (88). The elevation of serum [K+] to > 5.5 or > 6.0 mmol/L occurs gradually over time and may occur months or years after starting finerenone, depending on the declining kidney function and/or increasing serum [K+] (91, 96). In this regard, potassium monitoring should be performed at each clinical follow-up visit along with consideration of other conditions or triggers of a hyperkalemia event (i.e., medications like trimethoprim, volume depletion, acute illness, and AKI) (88, 96, 97). Overall, the potassium management algorithm and serum [K+] monitoring schedule described in the finerenone phase 3 study protocol, may serve as a framework for use in clinical practice, which is aligned to current guidelines and based on consideration of patient characteristics (i.e., eGFR and baseline serum [K+]) that may increase their risk of hyperkalemia (34, 88).

Finerenone induces a low absolute risk of clinically relevant hyperkalemia in DKD patients, which is manageable with temporary treatment interruption and dose reduction or use of other strategies such as co-treatment with diuretics, SGLT2i and new generation potassium binders (patiromer and sodium zirconium cyclosilicate) in the event of hyperkalemia detected on routine potassium monitoring (7, 8, 22, 88, 98).

## 8 Evidence from meta-analyses on the efficacy and safety of finerenone in patients with CKD and T2D

Table 1 summarizes the results of the meta-analyses of RCTs on the efficacy and safety of finerenone in patients with CKD and T2D, in finerenone vs. placebo/other non-steroidal MRAs (99–108) and finerenone vs. SGLT2i or GLP-1 RA (9, 109–111) comparisons.

**TABLE 1:** Meta-analyses on the efficacy and safety of finerenone in patients with CKD and T2D.

Patients with chronic kidney disease and type 2 diabetes			
Meta-analysis	RCTs (*n*)	Finerenone vs. SGLT2i and/or GLP-1 RA	Overall benefits of finerenone
Zheng et al. (99)	4 RCTs (*n* = 13,945)	Finerenone significantly reduced * UACR (MD −0.30; 95% CI: −0.33 to −0.27) * decrease in eGFR by 40% from baseline (RR 0.85; 95% CI: 0.78–0.93). Safety * same risk of AEs with placebo (RR 1.00; 95% CI: 0.98–1.01). * higher incidence of hyperkalemia than placebo (RR 2.03; 95% CI: 1.83–2.26)	Reduction of UACR Amelioration of the deterioration of renal function Higher risk of hyperkalemia but same risk of overall AEs.
Jyotsna et al. (100)	7 RCTs (*n* = 39,995)	Finerenone significantly decreased risk of * cardiovascular mortality (RR 0.86; 95% CI: 0.80–0.93), * renal-related mortality (RR 0.56; 95% CI: 0.17–1.82) Safety * a marginally reduced risk of serious adverse events (RR 0.95; 95% CI: 0.92–0.97) * similar overall risk of AEs (RR 1.00; 95% CI: 0.99–1.01)	Reduction of the risk of ESKD and renal failure Reduction of the risk of cardiovascular mortality and hospitalization
Bao et al. (101)	4 RCTs (*n* = 13,510)	Finerenone significantly reduced * UACR mean ratio (MD −0.30; 95% CI: −0.32 to −0.28) * decrease in eGFR by 40% from baseline (RR 0.85; 95% CI: 0.78–0.93) * ESKD (RR 0.80; 95% CI: 0.65–0.99) * cardiovascular events (RR 0.88; 95% CI: 0.80–0.96) Safety * similar all-cause mortality (RR 0.90; 95% CI: 0.80–1.00), and the incidence of AEs (RR 1.00; 95% CI: 0.98–1.01) * significantly higher incidence of hyperkalemia (RR 2.03; 95% CI: 1.83–2.26).	Significant renal and cardiovascular benefits without unacceptable side-effects.
Yasmin et al. (102)	7 RCTs (*n* = 15,462)	Finerenone significantly reduced * risk for cardiovascular mortality (HR 0.84; 95% CI: 0.74–0.95) * risk for heart failure (OR 0.79; 95% CI: 0.68–0.92) * decrease in eGFR by 40% (OR 0.82; 95% CI: 0.74–0.91) * decrease in eGFR by 57% (OR 0.70; 95% CI: 0.59–0.82) Safety * higher incidence of moderate hyperkalemia (OR 2.25; 95% CI: 1.78–2.84).	Reduction of the risk of heart failure and cardiovascular mortality Delayed progression of CKD A higher risk of hyperkalemia but rarely severe enough to merit treatment discontinuation
Abdelazeem et al. (104)	3 RCTs (*n* = 13,847)	Finerenone significantly decreased * rate of cardiovascular events (RR 0.88; 95% CI: 0.80–0.96, which was mainly driven by lower hospitalizations for heart failure (RR 0.79; 95% CI: 0.66–0.94) Similar to placebo in terms of * cardiovascular death (RR 0.88; 95% CI: 0.76–1.02) * non-fatal myocardial infarction (RR 0.91; 95% CI: 0.74–1.12) * non-fatal stroke (RR 0.99; 95% CI: 0.80–1.22).	Reduction of cardiovascular events and HHF
Ghosal et al. (105)	4 RCTs (*n* = 13,943)	Finerenone significantly reduced * UACR (SMD −0.49, 95% CI: −0.53 to −0.46) * decline in eGFR (SMD −0.32, 95% CI: −0.37 to −0.27) * with 16% reduction in the renal composite (kidney failure, decrease in eGFR by 40% from baseline or death from renal causes) (HR 0.84, 95% CI: 0.77–0.92) Safety * same risk of AEs with placebo (RR 1.00; 95% CI: 0.98–1.01) * increase in hyperkalemia (RR 2.22; 95% CI: 1.93–2.24)	Significant benefits in renal outcomes with a side effect profile comparable to placebo.
Yang et al. (106)	4 RCTs (*n* = 13,943)	Finerenone showed a great benefit in reducing the incidence of * MACE (RR 0.88; 95% CI 0.80–0.96) * all-cause mortality (RR 0.89; 95% CI: 0.80–0.99) * myocardial infarction (RR 0.79; 95% CI: 0.67–0.92) * new-onset hypertension (RR 0.71; 95% CI: 0.62–0.81) * no increase in cerebrovascular events and new-onset AF Safety * Same risk of total AEs with placebo (RR 1.00; 95% CI: 0.98–1.01) * Higher risk of hyperkalemia than placebo (RR 2.04; 95% CI: 1.80–2.32).	A great benefit of reducing the risk of MACE, all-cause mortality, myocardial infarction, and new-onset hypertension events
Zhu et al. (107)	7 RCTs (*n* = 15,618)	Finerenone significantly reduced * death from any cause (95% CI: 0.82–0.99) * risk of heart failure (95% CI: 0.67–0.92) Finerenone could not reduce the incidence of * cardiovascular mortality * myocardial infarction * hospitalization for any cause Safety * same risk of total AEs with placebo * higher risk of study-drug-related AEs (95% CI: 1.27–1.48).	Reduction in the risk of death from any cause and heart failure but a concomitant increase in the study-drug-related AEs
**Meta-analysis**	**RCTs (*n*)**	**Finerenone vs. placebo or other non-steroidal MRAs**	**Overall benefits of finerenone**
Jiang et al. (103)	8 RCTs (*n* = 14,450)	Non-steroidal MRAs versus placebo * a greater reduction in UACR (WMD −0.40, 95% CI: −0.48 to −0.32), * eGFR (WMD −2.69, 95% CI: −4.47 to −0.91) * systolic blood pressure (WMD −4.84, 95% CI: −5.96 to −3.72) Safety * similar incidence of SAEs (RR 1.32, 95% CI: 0.98–1.79) * a higher risk of hyperkalemia (RR 2.07, 95% CI: 1.86–2.30) Finerenone vs. apararenone and esaxerenone * similar to esaxerenone in UACR reduction (WMD 0.24, 95% CI: −0.016 to 0.496); * apararenone and esaxerenone showed greater decreases in SBP (WMD 1.37, 95% CI: 0.456–2.284 and WMD 3.11, 95% CI: 0.544–5,676, respectively)	Non-steroidal MRAs reduce proteinuria and SBP despite the moderate increased risk of hyperkalemia, In terms of renoprotection, esaxerenone and finerenone may have similar effects. Esaxerenone and apararenone may have better antihypertensive effects than finerenone.
Dutta et al. (108)	7 RCTs (*n* = 13,783)	Finerenone significantly revealed * greater chance of lowering of UACR from baseline at 90 days (MD 23.82%; 95% CI: −24.87 to −22.77), after 2 years (MD 37.9%; 95% CI: −38.09 to −37.71) and 4 years (MD 25.20%; 95% CI: −25.63 to −24.77) of treatment. * lower risk of > 40% decline in GFR (OR 0.83; 95% CI: 0.75–0.92). * lower risk of cardiovascular death, non-fatal myocardial infarction, non-fatal stroke or hospitalization for heart failure, as compared to placebo/eplerenone (OR 0.86; 95% CI: 0.78–0.95) Safety * TAEs was similar (RR 0.97; 95% CI: 0.88–1.07) * SAEs significantly lower (RR 0.91; 95% CI: 0.84–0.97) vs. controls	Beneficial effects in reducing UACR and GFR decline
**Meta-analysis**	**RCTs (*n*)**	**Finerenone vs. SGLT2i and/or GLP-1 RA**	**Overall benefits of finerenone**
Zhang et al. (9)	18 RCTs (*n* = 51,496)	Finerenone vs. placebo significantly reduced * risk of MACE (RR 0.88; 95% CI: 0.80–0.97), * renal outcome (RR 0.86; 95% CI: 0.79–0.93) * HHF (RR 0.79; 95% CI: 0.67–0.92) SGLT-2i vs. placebo significantly reduced * risk of MACE (RR 0.84; 95% CI: 0.78–0.90), * renal outcome (RR 0.67; 95% CI: 0.60–0.74) * HHF (RR 0.60; 95% CI: 0.53–0.68) * all-cause death (RR 0.89; 95% CI: 0.81–0.91) * Cardiovascular death (RR0.86; 95% CI: 0.77–0.96) GLP-1 RA vs. placebo significantly reduced * risk of MACE (RR 0.86; 95% CI: 0.78 to 0.94). SGLT2i had significant effect in renal outcome and HHF * vs. finerenone renal outcome (RR 1.29; 95% CI: 1.13–1.47] * vs. finerenone HHF (RR 1.31; 95% CI: 1.07–1.61) * vs. GLP-1 RA renal outcome (RR 1.36; 95% CI: 1.16–1.59) * vs. GLP-1 RA HHF (RR 1.49, 95% CI: 1.18–1.89)	SGLT2i, GLP-1 RA and finerenone are comparable in MACE, ACD and CVD SGLT2i significantly decreases the risk of renal events and HHF compared with finerenone and GLP-1 RA.
Gu et al. (109)	11 RCTs (*n* = 73,927) vs. GLP1-RA	Finerenone and GLP1 RA were similar in * reducing the risk of kidney disease progression (HR 0.84; 95% CI: 0.77–0.92 for finerenone and HR 0.81; 95% CI: 0.76–0.86 for GLP1-RA) * reducing the risk of MACE by 13% (HR 0.87; 95% CI: 0.79–0.95 for finerenone and HR 0.87; 95% CI: 0.83 to 0.92 for GLP-1 RA), in patients with established atherosclerotic cardiovascular disease GLP1-RA was superior to finerenone in * reducing myocardial infarction, stroke and cardiovascular death Finerenone was superior to GLP-1 RA in * in delaying deterioration of kidney function Finerenone was beneficial for * reducing the risk of HHF (HR 0.78; 95% CI: 0.66–0.92)	Finerenone and GLP1-RA are similar in terms of risk reduction in MACE and lowering the risk of progression of kidney disease, Only finerenone has a significant protective effect against HHF
Zhang et al. (110)	10 RCTs (*n* = 35,841) vs. SGLT2i	Finerenone vs. placebo * a decreased risk of AF (RR 0.79, 95% CI: 0.62–0.99) SGLT2i vs. placebo * no effect on the risk of AF SGLT2i vs. finerenone * a decreased risk of HHF (RR 0.78, 95% CI: 0.63–0.98) Finerenone and SGLT2i were similar in terms of * AF (RR 0.84, 95% CI: 0.48–1.46) * MACE (RR 0.93, 95% CI: 0.81–1.06) * nonfatal stroke (RR 0.78, 95% CI: 0.58–1.05). * no significant risk of AEs compared with placebo	Finerenone and SGLT2i are similar in terms of the reduction of new-onset of AF
Zhao et al. (111)	14 RCTs (*n* = 13,246) vs. gliflozins	Gliflozins vs. finerenone greater reduction in the risk of * progression to ESKD (HR 0.78, 95% CI: 0.67–0.90) * HHF (HR 0.71, 95% CI: 0.55–0.92)	Superiority of gliflozins over finerenone in preventing renal failure and heart failure (may be a class effect valid only for some gliflozins)

AF, Atrial fibrillation; AE, adverse event; CI, Confidence interval; eGFR, Estimated glomerular filtration rate; ESKD, End-stage kidney disease; HR, Hazard ratio; HHF, Hospitalization for heart failure; OR, Odds ratio; MACE, Major adverse cardiovascular events; MD, Mean difference; RR, relative risk; SAEs, Serious adverse events; SBP, Systolic blood pressure; RCTs, Randomized controlled trials; TAEs, Treatment emergent adverse events; UACR, Urinary albumin-to-creatinine ratio; WMD, weighted mean difference.

### 8.1 Finerenone vs. placebo or other non-steroidal MRAs

Overall, the meta-analyses confirmed the renal benefits of finerenone (vs. placebo) in patients with T2D and CKD in terms of the reducing the UACR, ameliorating the deterioration of renal function with reduced risk of ESKD and renal failure (99–102, 105, 108) (Table 1).

Majority of meta-analyses confirmed the cardiovascular benefits of finerenone (vs. placebo) in terms of reducing the risk of cardiovascular events (101, 104, 106), new-onset hypertension events (106), HHF (100, 102, 104, 107, 108), myocardial infarction (99, 106, 108), cardiovascular mortality (100, 102, 108) and all-cause mortality (106, 107). However, some meta-analyses revealed that cerebrovascular events and new-onset atrial fibrillation also did not increase in patients taking finerenone (106), while others reported no significant differences between finerenone and placebo groups in terms of cardiovascular death, non-fatal myocardial infarction and non-fatal stroke (104) and that finerenone could not reduce the incidence of death from cardiovascular, myocardial infarction and hospitalization for any cause (107) (Table 1).

Considering the safety, majority of meta-analyses revealed a higher risk of moderate hyperkalemia in the finerenone group compared with placebo but no difference in the risk of overall adverse events (99–103, 105, 106), while some meta-analyses also reported a marginally reduced risk of SAEs (100) as well as a higher risk of study-drug-related advent events (107) (Table 1).

The meta-analyses comparing finerenone vs. other MRAs revealed similar renoprotective effects of esaxerenone and finerenone (103), better antihypertensive effects of esaxerenone and apararenone than finerenone (103) and lower occurrence of cardiovascular death, non-fatal myocardial infarction, non-fatal stroke or hospitalization for heart failure with finerenone vs. eplerenone (108) (Table 1).

### 8.2 Finerenone vs. GLP1-RA and/or SGLTi

The meta-analyses of finerenone (vs. GLP1-RA and/or SGLTi) in patients with CKD and T2D revealed controversial findings (Table 1), including:

• SGLT2i, GLP-1 RA and finerenone were comparable in MACE, ACD and CVD, while SGLT2i significantly decreased the risk of renal events and HHF compared with finerenone and GLP-1 RA (9).
• Finerenone and GLP1-RA were similar in terms of a risk reduction in MACE, whereas only finerenone had a significant protective effect against HHF (109).
• Both finerenone and GLP1-RA reduced the risk of kidney disease progression, including macroalbuminuria, but finerenone was superior to GLP1-RA in delaying deterioration of kidney function (109).
• Finerenone and SGLT2i were comparable in AF, MACE and nonfatal stroke but SGLTi was associated with a decreased risk of HHF (RR 0.78, 95% CI 0.63–0.98) and both showed no significant risk of adverse events compared with placebo (110).
• Gliflozins were superior over finerenone in preventing renal failure and heart failure events and the possibly in preventing atherosclerotic cardiovascular and death endpoints (111).
• Finerenone but not SGLT2i was associated with a decreased risk of AF compared with placebo, while SGLT2i were associated with a decreased risk of HHF compared with finerenone. Finerenone and SGLT2i were comparable in AF, MACE and nonfatal stroke and they both showed no significant risk of adverse events compared with placebo (110).

### 8.3 Clinical relevance of data from the finerenone-based meta-analyses

Accordingly, finerenone is considered a promising therapeutic tool in T2D patients with established CKD, given the significant benefits in renal outcomes in terms of reducing the UACR and GFR decline and the risk of ESKD and renal failure with a side effect profile comparable to placebo (100, 101, 105) Finerenone, owing to its better mineralocorticoid affinity, and a much lower risk of adverse effects, is suggested to be a much better alternative than other RAS blockers available for the treatment of CKD patients with T2D (102).

Its potential cardiovascular benefits such as reducing new-onset hypertension events, MACE, HHF, myocardial infarction and cardiovascular mortality in patients with CKD and T2D are also notable (99–102, 104, 106–108). However, while the SGLT2i, GLP-1 RA and finerenone are considered comparable in their effects on reducing the MACE, HHF and cardiovascular death, their absolute benefit is suggested to vary in each patient depending on baseline risks for cardiovascular and kidney outcomes, emphasizing that the treatment decisions should consider the clinical benefit profiles of each drug (9, 109, 110, 112).

## 9 Conclusion

The evolving data regarding the efficacy of SGLT2is and non-steroidal MRAs on slowing CKD progression and reducing CV risk seem to provide the opportunity to use pillars of therapy in managing DKD, after a long-period of treatment scarcity in this field. In this regard, by combining RAS blockade with SGLT2i, GLP-1 RA and finerenone, clinicians seem to have a chance to address several key factors (hemodynamic dysfunction, inflammation and fibrosis, optimal glycemic control) implicated in the progression of DKD to enable a better prognosis and slower disease progression.

Finerenone slows CKD progression, reduces albuminuria, and prevents CV complications, regardless of the baseline HbA1c levels and concomitant treatments (SGLT2i, GLP-1 RA or insulin), with a favorable benefit-risk profile. Hence, the emergence of finerenone as a new therapeutic tool, and the recognition of the albuminuria as a powerful marker to detect patients at high risk of cardiorenal disease, are important developments that would likely to impact standard-of-care options in the setting of DKD.

Nonetheless, future research is warranted to better understand the cardiorenal benefits offered by finerenone in diabetic and non-diabetic CKD population, to clarify the mechanisms of action and to verify the possible synergistic effect of finerenone co-treatment with SGLT2i and GLP-1 RAs.

Ongoing trials addressing the efficacy of finerenone on the rate of change in the eGFR slope from baseline in patients with non-diabetic CKD (FIND-CKD trial, NCT05047263), the safety and efficacy of finerenone in children and adolescents with CKD and severely elevated proteinuria (FIONA trial), the levels of biomarkers of pathological processes (inflammation, fibrosis, vascular function, and congestion, add-on FIGARO-BM trial, NCT05013008), and the potential benefit of combining empagliflozin and finerenone in UACR reduction in DKD patients (CONFIDENCE trial, NCT05254002) (12, 83) seem to provide further mechanistic insights on the effects of finerenone, and to help clinicians to optimize the positioning of this new drug within the current DKD management landscape.

## Author contributions

MArici: Conceptualization, Investigation, Methodology, Project administration, Writing – original draft, Writing – review & editing. BA: Project administration, Writing – original draft, Writing – review & editing, Conceptualization, Investigation, Methodology. MAra: Conceptualization, Investigation, Methodology, Project administration, Writing – original draft, Writing – review & editing. AA: Conceptualization, Investigation, Methodology, Project administration, Writing – original draft, Writing – review & editing. TD: Conceptualization, Investigation, Methodology, Project administration, Writing – original draft, Writing – review & editing. TE: Conceptualization, Investigation, Methodology, Project administration, Writing – original draft, Writing – review & editing. GG: Conceptualization, Investigation, Methodology, Project administration, Writing – original draft, Writing – review & editing. DG: Conceptualization, Investigation, Methodology, Project administration, Writing – original draft, Writing – review & editing. AY: Conceptualization, Investigation, Methodology, Project administration, Writing – original draft, Writing – review & editing. TY: Conceptualization, Investigation, Methodology, Project administration, Writing – original draft, Writing – review & editing.
